# What Do Survivors of Child Sexual Abuse Believe Will Facilitate Early Disclosure of Sexual Abuse?

**DOI:** 10.3389/fpsyt.2021.639341

**Published:** 2021-06-14

**Authors:** Ellen Tvedt Solberg, Jorunn E. Halvorsen, Signe Hjelen Stige

**Affiliations:** ^1^Department of Child and Adolescent Psychiatry, Helse Fonna, Haugesund, Norway; ^2^Department of Health and Welfare Services, Municipality of Bergen, Bergen, Norway; ^3^Department of Clinical Psychology, University of Bergen, Bergen, Norway

**Keywords:** child sexual abuse, disclosure, facilitation, qualitative interview, thematic analysis

## Abstract

The purpose of this study was to explore what adult survivors of child sexual abuse (CSA) believe will facilitate early disclosure of sexual abuse. We conducted semi-structured interviews with 12 adult survivors of CSA aged 18–57 years, and analyzed the transcripts using reflexive thematic analysis. Our analysis resulted in three main themes: *Adults making it possible to tell; Adults daring to consider the unthinkable;* and *Conveying knowledge of CSA that facilitates understanding*. The findings highlighted the importance of adults facilitating disclosure and making it possible for survivors to tell about their experiences, but also the responsibility adults have to dare to consider the possibility of CSA when children struggle. Another important finding was the significance of enhancing the general population's understanding of the complexity of CSA, including why disclosure is difficult, so adults are given the tools needed to understand and know what to do when they suspect CSA. The findings also have important implications for the planning and implementation of interventions to facilitate early disclosure of CSA.

## Introduction

Approximately 15–18% of women and 6–7.6% of men experience CSA (Child sexual abuse) (1–3). On average it can take up to 18 years before victims disclose CSA (4–6), some studies showing a small number (8–21%) disclose after 1 month (6–8), and around 27.8% never disclose (9–11). The health consequences have shown to be severe, both for the physical and mental health of victims (4, 8, 12, 13). CSA has proven to have more serious consequences than other negative experiences during childhood (14–16), and sexual abuse by primary caretakers can lead to maladaptive normal development (5, 17).

There is a discrepancy between the number of reported cases and incident of CSA documented through self-reports (1, 2, 7, 18, 19). This indicates that many survivors never talk about their experiences to the public health services, and some never tell anyone about their experiences of CSA (9–11). How to facilitate early disclosure of CSA is therefore an important goal, both in order to secure children that experience ongoing abuse, but also because the negative outcomes caused by these adverse childhood experiences have been shown to increase by the number of experiences. Early disclosure may therefore help prevent sexual abuse from being repeated, and prevent more serious health issues in the future (8, 12, 20–22).

However, facilitating disclosure is not easy due to personal and intra-personal barriers, frequently reported by victims (7, 20, 23). Many children do not self-label their experiences as sexual abuse (24, 25), others feel a strong ambiguity toward the perpetrator, especially if this is a primary caretaker (5, 24, 26–28), or they experience feelings of fear, shame and guilt (29–31). Age and gender has also been found to influence this process, with some studies showing that older children disclose their experiences more easily (4, 13), other studies report that children are most likely to disclose at the age when the abuse has happened and that all ages therefore should be targeted for prevention (32). The severity of the sexual abuse and a supportive primary caregiver has shown to increase the likelihood of disclosure (13, 25).

Most victims disclose to their friends or parents first (7, 25). Some studies show that children often tell their parents, while adolescents, not wanting to burden parents, more often tell their friends (11, 29, 33, 34). Moreover, children report that they often test and interpret how others react to what they say and do, and that this influences their disclosure (35, 36).

The timing and manner in which one asks about sexual abuse has also shown to be important in facilitating disclosure, specifically the need to provide safe contexts (20, 37). Children and youth often underline how difficult it can be to find an appropriate moment to talk about their experiences, and adults somehow facilitating these situations could make it easier (20, 35, 38). Parents on their side report difficulties talking to their children about potential CSA not knowing what to say, and many still hold misconceptions about CSA such as underestimating its prevalence (39, 40), thus making the facilitation children need more difficult.

While there has been made considerable efforts in the research community to develop improved methods of facilitating disclosure, this remains a highly complicated process. It has been underlined that spreading information and prevention programs can help in the process of facilitation, and also improve how we respond in a helpful way (8, 20, 37), but there are also findings suggesting that advanced focus can be a barrier to disclosure because it creates a stigma around victims, and that keeping quiet is a way of maintaining control and avoiding a negative self-representation (41). The aim of this article is therefore to add the perspectives of survivors of CSA, and what they believe can facilitate early disclosure, and that this might add insights to ongoing efforts to facilitate in the complex process of early disclosure.

## Methods

### Study Setting

This study was conducted in the second biggest city in Norway, in a societal context where the last years have seen an increased focus on disclosure of sexual abuse (e.g., police uncovering networks of cyber abuse, campaigns encouraging people to report suspicions of sexual abuse, “me too” - campaign). Historically, sexual abuse has been denied and silenced, and there is still a taboo related to sharing experiences of sexual abuse. In this study, for practical and ethical considerations, we have spoken to adults looking back on their experiences as children and youth, and the current findings should therefore be understood within this context.

### Research Design

The current study is a part of a larger project exploring the process and consequences of disclosing CSA. Two articles have previously been published from the study. One article explored the process leading survivors to understand that they had been sexually abused, with the main themes *The ambiguity of memory: “To remember the catastrophic”; The language of the body: “Let the body speak*”; and *Encountering an observant other: “The significance of being seen and recognized”* (42). Another article explored survivors' experienced barriers in their process of disclosing sexual abuse, with the main themes Fear of reprisals; CSA stains – Negative implications for self-representation; and The complicating effect of ambiguity (41). In this article, we explore adult survivors of child sexual abuse's reflections and perspectives on what could facilitate early disclosure of CSA. We invited the survivors to reflect upon what would facilitate processes of early disclosure of CSA using their own experiences. Their perspective as adult survivors of CSA is unique, and could give important insights to the field. The use of a qualitative research design allowed us to get close to the participants' own experiences and reflections, while at the same time reflecting on how our interpretations influence the data.

### Recruitment Procedure and Participants

In order to recruit participants to our research project, we distributed posters with information about the project to strategic locations. This included mental health out-patient clinics for adults and a support center for survivors of sexual abuse. The poster carried information that we were seeking adults who had experienced CSA, and that we wanted to interview them about the process of disclosing CSA. The poster also informed about the project being qualitative research, and that focus of the interviews would be on what they experienced made it difficult to disclose, what could make it easier, and what survivors of CSA need to disclose. The participants were included based on the criteria of having experienced CSA before the age of 18, and being older than 18 years old when included in the study. Interested participants first contacted the third author, who provided additional information about the project and did an initial assessment of basic criteria as established by the committee of ethics (i.e., competency to consent, no suicidal risk or ongoing psychotic episode). Twelve participants (nine women and three men) between the ages of 18–57 (average 36 years) contacted the third author, and all were included in the study. For five of the participants their perpetrator was primary caregiver, for two participants it was extended family members, and five participants had no family relationship to their perpetrator. All participants had already disclosed their experiences.

### Data Collection

The interviews were all carried out by the first and second author under supervision of the third author, who was on stand by in case participants got dysregulated during the interviews and were in need of support. There were no such incidents. The interviews were semi-structured, allowing the interviewers to follow the participants' story, while at the same time maintaining a focus on the intended aim of the project; what the participants believed could facilitate disclosure of CSA. The interviewers would seek clarification by asking for examples, trying to understand the participants' experiences more correctly (see Appendix). The interviews lasted between 60 and 105 min and were transcribed verbatim by the interviewer. CSA was not measured systematically before or during the interviews, but defined by the participants themselves as something they had experienced. This was done in line with a qualitative design where the experience of the informants is highlighted.

### Data Analysis

When analyzing the interviews, we used reflexive thematic analysis as described by Braun and Clarke (43, 44), highlighting exploration, the perspective of the participants, the researchers' interpretations, and reflexivity (45). As technical support we used NVivo 12 (46). The analytical process was carried out through several steps as described by Braun and Clarke (43, 44). First every author familiarized themselves with every transcript, and then the analytical focus was decided; What do survivors of CSA believe will facilitate early disclosure. The first author coded the material according to the analytical focus of this article, highlighting what stood out as interesting through an explorative and inductive approach. Then all authors familiarized themselves with the codes and met to start the process of group the coded material together across the interviews, constructing a first, tentative theme structure. The first author then refined the final thematic structure, before consensus was reached on the three themes given in this article; *Adults making it possible to tell; Adults daring to consider the unthinkable;* and *Conveying knowledge of CSA that facilitates understanding*. Through the whole process of analysis all three authors frequently consulted the interview transcripts to ensure that details didn't get lost. Throughout the process, the perspectives of an explorative and inductive attitude to the material, and reflection around our influence as researchers, was continuously discussed.

### Ethics

Our participants were all survivors of CSA and therefore part of a vulnerable group particularly prone to physical and mental health issues. To maintain the focus on their emotional well-being throughout the study and treating the material with respect, were therefore important aspects. We have throughout the process continued to discuss ethical dilemmas, such as considerations with the use of words (“survivor” instead of “victim”), and asking in an open way about their experiences without inquiring too many details.

We did not use health status as inclusion or exclusion criteria, but we considered every participant as part of a vulnerable group and took precautions accordingly. The regional committee for ethics in medical research (Rek Vest – approval number 2017/1623/REK Vest) approved the study with the condition of the third author (psychologist with clinical specialty) being in proximity during interviews and that the third author also made an initial assessment before the participants were included in the study. The interviewers underwent training in reading signs of dysregulation and in how to intervene, and had knowledge about trauma psychology and some clinical experience. The third author was available during every interview, but there were no incidents with the need to intervene. We also made sure that the participants did not leave the interview dysregulated, and closed the session by talking about easier subjects.

Throughout the study we aimed to take care of the participants in a respectful way. We considered this as important with a vulnerable group, and we were aware and reflected upon them not feeling forced to participate. The participants joined voluntarily on their own initiative and could at any time withdraw from the study. No participants used this option. Moreover, all the participants seemed motivated for participation, shared rich descriptions of their experiences and perspectives during interviews, and several participants commented that their participation contributed to a feeling of taking control over their trauma, and that it gave them meaning to share experiences and contribute to spreading knowledge about CSA.

### Reflexivity

Reflexivity remained an important topic throughout the research process through acknowledging our influence on the research process, including data collection and interpretation of the data material (43, 47). Psychological trauma and the way it impacts survivors was a shared interest by all three authors, leading to the initiation of this study. We also shared an interest in how and when mental health services can be helpful in a survivor's process. These interests may have made us more prone to pursue these particular aspects of the participants' experiences, but it has also ensured engagement with the phenomenon being studied. The three authors have different professional backgrounds; The first author recently completed her training as a clinical psychologist, the second author is a social worker who also just finished training as a clinical psychologist, and the third author is an associate professor and clinical psychologist. Throughout the process, we have sought to enhance our own consciousness about the way our backgrounds influence our understanding, utilizing the opportunities working as a researcher team provides to clarify our own perspectives and preunderstandings. We have consciously worked to enter an open and explorative attitude during interviews, reading of transcripts, analyzes and writing. One example is how one participant emphasized a downside to conveying knowledge about CSA to children because this could force them into an adult world, where we previously considered this as exclusively positive.

## Results

### Adults Making It Possible to Tell

An important theme that evolved throughout our analysis was the central role adults can play in disclosing sexual abuse. The participants stressed the significance of being asked directly about abuse, the impact of the relationship to the one asking, the timing for being asked as well as the need for a safe environment.

Almost all the participants pointed out that being asked was an important facilitator to disclosure. Most of them were never asked directly when they were younger, and were not sure if they would have been able to confirm if asked. However, they reflected on how it could have started a process in them, had they been asked at an earlier point.

*I: […] If somebody had asked you when you were younger if something had happened to you, “Has someone done this or that”, what do you think you would have said?* Well, maybe some things could have started to bubble up earlier. *I: If someone had asked?* Yes, I think direct questions without going around anything. I think that's the best. (Man, 50s)

Participants had, however, different perspectives on how adults should act when probing for CSA. While one participant stressed the importance of not to stop asking a child about sexual abuse even if the children initially deny, other participants underlined the importance of not pressing too hard for disclosure, arguing that it may actually make things worse. One participant described that being forced to tell felt like being pushed to jump into water from 10 meters height when not wanting to.

The participants also reflected on the significance of who asks the child. Most participants emphasized that in order to disclose, children need to feel trust in a relation, while taking the time required in order to establish a feeling of safety. Significantly, most participants did not disclose to their parents first, independent of their perpetrators being in the family or not. Some reflected that the reasons for this might have been that they were not used to sharing intimate details in their family, but also that some things are easier to discuss with someone who is not that close in relation. One participant explains:

I think it can be very difficult to talk to your parents, and I probably didn't say anything to my doctor because my parents were there. It could have been easier with a school nurse since they are not that close, and even easier with friends because they are younger. Somehow keeping it away from the people you are closest to. (Woman, 20s)

Our participants also reflected on the importance of timing and space, but also the feeling that it was a person who they could trust, and who was genuine.

What I had experienced earlier with therapists, psychologists and psychiatrists was that they sit and almost protect themselves behind a big desk or talk and talk and talk and don't dare to do anything else. […] yes, so he [the therapist which the participant finally disclosed to] could be personal, talk about his own life and this made us more at the same level *I: Okey, so that helped somehow that he*. Yes he was much more trustworthy than the others I had seen earlier. (Man, 50s)

### Adults Daring to Consider the Unthinkable

Most participants discussed how important it is that adults dare consider the unthinkable; that CSA happens. They stressed how adults need to take a larger responsibility and acknowledge that CSA happens, and not just place the responsibility of disclosure on the children. The participants spoke about their experiences with adults misinterpreting or overlooking important signs that they expressed as children and youth. However, they also reflected on a dilemma adults face when suspecting someone of CSA; fearing both to be right and wrong.

It has such big consequences [to accuse someone of CSA], and it's extremely uncomfortable. If one is going to talk about these tings, to take these tings seriously, then of course you think about “what if I'm wrong?”. What have you done then, what have you started? (Woman, late adolescence)

Many participants shared the experience that some adults, either in school, the community or family, were worried and suspected that something was happening. Some took action, but not enough. Some didn't do anything. In retrospect, participants reflected on the importance of adults taking a more active part and daring to ask and understand what they see.

And at that time I was in a choir, and I have kind of understood that many of the choir leaders and teachers reacted, and…. *I: Do you know what they reacted to?* It was probably just the change, I've heard that I had a hard look on my face, and kind of, everything kind of, my eyes were completely empty and, well I lost a lot of weight. [….] *I: You said your parents understood that something was wrong, the choir leader understood something was wrong, at school they understood something?* Yes they understood something, but they did nothing. (Woman, 20s)

Some of the participants recounted how they put on a happy face, performed well in school, kept silent and tried to go unnoticed. They highlighted how not only the children with disruptive behavior should be noticed, but also the silent ones:

I have always been good at hiding you know. *I: Ok. Always been the one who is fine, or how did you hide it?* Acting. *I: Ok, what did you act? Someone happy or?* Kind of, fake it till you make it. *I: And what did you fake?* That nothing happened. *I: So you just played a normal, happy 3, 4, 5, 6, 7 – year old?* Yes. (Woman, 20s)

According to many participants, their difficulties and changing behavior were interpreted as caused by other things such as puberty, parents' divorce or bullying. They underlined how adults must dare to look beyond behavior and try to see the signs that children and youth give. To be able to do this, people need to know what to look for:

Well, later on I've been very angry because there were so many red flags. *I: Can you talk a little about the red flags?* […] In sixth grade I got a lot of bruises and I completely changed my personality and I started seeing a psychologist, but since my parents had just divorced everyone thought that was the reason for my behavior. And then I started self-harming, not with a knife but scratching myself. And my mom has told me later that there was an extreme number of red flags and symptoms, but she just, it just didn't occur to her. (Woman, late adolescence)

### Conveying Knowledge of CSA That Facilitates Understanding

The participants all emphasized how important it is to convey knowledge to facilitate disclosure of CSA. They described the importance of openness and information, both in what to look for, but also what to do with suspicions about sexual abuse.

Due to the age range, the participants grew up in slightly different societies and contexts regarding the view and focus on CSA. The older participants talked about how the society has changed during their lifetime, how when they were younger, no one talked about sexual abuse, and people kept things to themselves. Almost all of the participants underlined that the openness in today's society constitutes a major step toward fighting taboos around sexual abuse, and most of them see a huge improvement from just a few years back.

It was highlighted by most participants that information conveyed to children, youth and adults is a key to facilitate disclosure. Some also pointed out how openness about feelings is important, especially which feelings are normal after experiencing sexual abuse. Some talked about the importance of not only learning what sexual abuse is, but also sexual education from an early age in order to learn what is normal and what is not:

My process started with learning about what sex really is, because you don't learn about it until late elementary school. So I didn't understand what had happened, and when you then learn what has happened, and they tell you how important it is to wait until you are ready and stuff like that, then you get that feeling: yeah, but I can't wait, because it has already happened, and, it's kind of, to go through that process and to know that someone took something away from you without you knowing they did. (Woman, 20s)

Teaching children about the limits to what adults may do and may not do, where they can touch and not, was considered important. However, one participant underlined that we have to be careful not to force an adult world upon children:

What I am afraid of, I've been thinking, that I don't want to spread my fear to my kids, you know, in new areas, because they're supposed to be children and fearless. That's what's natural, that children grow up with adults who don't do bad things. *I: That takes care of them and…* yes, let's them be themselves you know. (Man, 50s)

All participants emphasized the importance of informing adults about which signs to look for, because it cannot be up to children or youth alone to disclose something as difficult as sexual abuse. Several participants also underlined the need to convey what people should do if they suspect CSA. A few of the participants talked about how their caretakers suspected something, but they didn't know what to do or who to talk with:

It could have helped if my parents in some way knew who to contact, how to move forward, because I think that it's almost nobody who thinks that “my kid will be exposed to sexual abuse”. Well, sometimes there are parents who do it themselves, but most people don't think, you know. “What do I need to do if it happens”, right? *I: So we should have almost like an instruction manual saying “what to do if I suspect my child has been exposed to sexual abuse”?* Yes, what to do and that we have a system that can receive caretakers, schools and anybody who suspects anything. Because I think my parents often have felt rejected and very, like, just let things pass, you know. (Woman, 20s)

## Discussion

In this study, through the perspective and information given by survivors of CSA, we have gained insights into how early disclosure might be facilitated. One of the most important findings in our interviews points to the important role adults play in early disclosure of CSA. Previous research has shed light on the number of barriers survivors of CSA face in the process of disclosure (20, 23, 25, 41). Telling someone about CSA is therefore a complex and heavy process that can take years for many victims (4, 5, 37). This line of research highlights the importance of others contributing to making early disclosure of CSA possible. That adults should carry much of the responsibility for uncovering CSA seems so obvious it should hardly be a finding in an empirical article. Yet, many who experience CSA change behavior [e.g., get restless, and have emotional outbursts such as anger or self-harm, (16, 31, 42)], indicating to their surroundings that something is not right, without adults noticing (1, 2, 18, 19), or misinterpreting these signs (36, 42). Why, then, do adults around victims so often seem to either not understand or overlook the signs that children and youth experiencing CSA provide?

Child sexual abuse shatters our basic assumptions about the world as meaningful and benevolent others (28). Holding on to these assumptions are in many ways important for our survival. People are therefore hesitant to change these beliefs because they provide stability and help form attachments and trust in others. The way CSA contradicts basic assumptions makes it prone to be overlook or disregarded. These processes are strengthened by the way accepting that good and evil coexist can appears more difficult and frightening than just taking in evil from a distance (48). This is also reflected in research showing that people often underestimate the prevalence of CSA, think of most perpetrators as strangers, and find it difficult to consider that sexual abuse might happen to someone close to them (39, 40). This shows how people can be hesitant to even just consider sexual abuse when it happens close to them and are prone to write off claims or suspicions of wrong doings, like sexual abuse – thus why so many survivors of CSA experience that their signs that something was wrong were overlooked or misinterpreted by the adults in their surroundings.

Given that we are hesitant to change our fundamental assumptions about a safe world, daring to consider CSA is something that takes great effort and elicits difficulties; Is it worse that the suspicions are right and the consequences that this cause, or to wrongly accuse someone of a terrible deed? Herman (17) claims that people often take the side of the perpetrator because it's the easier path. It's less difficult to claim that one person, a potential victim, is lying, than taking in the horrible possibility of the victim being right, thus forcing us to change our basic assumptions and accept that bad things can happen in our proximity. Studies in cognitive psychology have shown that people often take the easiest way to think and make decisions in order to avoid cognitive strain (49). When people stop thinking, stop paying attention and stop questioning things around them, it can become a barrier to disclosure of CSA (48). Given our findings of the key role adults play if earlier disclosure of CSA is to be possible, and the challenges outlined above, how to influence people, especially adults in contact with children, to think the unthinkable and act on their suspicions of CSA?

One aspect stressed by our participants was the importance of conveying knowledge about CSA. If one does not know what to look for, it is difficult to notice. Spreading information to adults in contact with children (teachers, health professionals, parents e.g.,) about common signs of CSA could help increase awareness. An important implication of our findings is therefore how giving adults the understanding of how difficult it is for survivors to disclose CSA, and at the same time give them the tools to recognize its physical and psychological effects could help facilitate earlier disclosure of CSA. This is in line with earlier research (7). This could also enhance the understanding of the responsibility adults have in the process of disclosure, precisely because of the barriers survivors face. It cannot be the responsibility of the child alone to stop the abuse. The increased focus in today's society and media on CSA might force us to consider and to a greater degree understand CSA, its prevalence and impact. Thus, conveying knowledge through educational programs, websites, posters, etc. aimed at adults could be important to facilitate early disclosure.

At the same time, daring to consider CSA does not alone facilitate disclosure. Our findings show that many participants experienced that relatives or other adults suspected that something was happening, but that they did not dare or know what to do. Thus, another important implication of our findings is the importance of teaching people how to act on their suspicions. It will therefore be important to make information and a support system more available, especially targeting adults with and working around children. Also, with some studies showing that many adolescents disclose to their peers (11, 29, 33, 34), this kind of information and support should also target this group. One possible intervention could be creating platforms with the possibility of discussing concerns and making information about what to with your suspicions more accessible. One example of efforts in this direction is the Norway government's official webpage (50), offering information and support online for survivors of violence and abuse, people suspecting abuse, and for perpetrators. It offers information about available treatment options and support services. However, the impact of this resource is uncertain, and it underlines the need to systematize efforts to facilitate early disclosure of CSA (42).

Our participants reflected on the difficulty of talking about something as personal and shameful as sexual abuse, and underline the significance of others initiating the conversation, and how important being asked, but also trust and timing is. Parents, but also health professionals, often report that they hesitate to ask about sexual abuse (51). Having a joint focus of attention, like a TV-program or a story targeting sexual abuse, has shown to help children or parents to ask or talk about sexual abuse. The joint focus serves as an indirect and less confrontative entry into a difficult conversation (20, 38). Based on this it can be assumed that todays' focus on children's programs and large media coverage on CSA might give more opportunities for a joint focus of attention. There is also evidence to show that educational programs increase communication between parents and their children (20, 40). Another implication of this research focus is then to create opportunities of joint attention and communication between parents and their children.

Conveying knowledge to children and youth about limits of intimacy, sex and what adults are allowed to do, is another implication stressed by our participants. They believe that giving children and youth a possibility and the vocabulary to understand what happens and where the limit goes is important. If they know that what happens is wrong, it can make it easier for them to disclose. It may also enhance resilience and empower children at risk to say “no” because they know that they are allowed and entitled to say no. This is in line with findings showing that children in all ages should be targeted for prevention programs and learn about CSA, because they are more likely to disclose at the age when the abuse has happened (32).

However, increased information about the long-lasting effects of CSA can also become a barrier for disclosure because it creates a stigma around victims, and that keeping quiet is a way of maintaining control and avoiding a negative self-representation (41). In our findings also another dilemma was raised with a concern that an exaggerated focus on CSA may force children into an adult world and putting too much of the responsibility to prevent and disclose abuse on the children's shoulders. In teaching children about serious topics and the importance of disclosing, it may make them more suspicious and force them to try to understand issues that are too complex for them. One could argue that children should be allowed to be children and play carefree, and that instead there should be an increased focus on the responsibility of adults.

Increasing knowledge about CSA and focusing on the responsibility of adults can thus be seen as the most important facilitator toward disclosure. However, with a large focus on conveying knowledge and forcing adults to acknowledge that CSA happens another important dilemma arises; If adults become too watchful and suspicious toward other adults, it might scare many away from a natural closeness with children. Close relationships and playing adults are shaping important aspects of the child (52). It is therefore important to maintain a balance so as not to suspect everyone, and not scare adults into keeping a distance from children to avoid eliciting suspicion of CSA. At the same time, it is essential that people dare to consider that bad doings can happen close to us. This shows the complexity and many difficulties surrounding the disclosure of CSA also for non-victims, and in order to facilitate disclosure these aspects need to be shed light on and discussed.

### Limitations

In the current study we used a qualitative design with a reflexive thematic analytical approach to analysis. In this methodology the influence of the researchers are highlighted, and this has been an ongoing focus throughout the study. There is a small sample of participants (12) in this study, which by some will be seen as a limitation. The focus has been on the understanding and experiences of the participants, and the aim to shed light on the complexities in the process.

While our pool of participants included both genders, we ended up interviewing more women than men. Further research should aim to recruit more men, especially given that men often experience different kinds of barriers to disclosure than women (53). There was also a variation in the relationship to the perpetrator among our participants. There might be different aspects that facilitate disclosure depending on the relationship and closeness between victim and perpetrator that our study fails to recognize.

All participants were raised in x, and their thoughts and reflections are influenced by the given society. As all knowledge is situated, our findings and implications are influenced by the cultural context in which the study is conducted and us as researchers. As a consequence, the transferability of the findings will be established by the readers and by future research, with our transparency on context and research process facilitating the readers' assessment of the trustworthiness and transferability of the presented findings (54).

Through the interviews the adult participants looked back to their childhood. There is often a difference in asking adults looking back on disclosure and asking children who have recently been through this process. When looking back as an adult they are at a different developmental stage, and events and decisions are often interpreted differently. One may argue that it is problematic to use knowledge from adults to enhance an understanding of facilitating disclosure among children. At the same time, it can be easier for adults to understand the perspective of other adults when trying to understand how to facilitate disclosure. But the retrospective design and age range of the participants is important to keep in mind when considering the transferability of the findings.

## Conclusion

The current study aimed to gain insights into how we might facilitate early disclosure of CSA by exploring the perspective and reflections of adult survivors of CSA. Through in-depth interviews with 12 survivors we found three main themes that highlight the importance of increased awareness and understanding of CSA and its complexities within the general population, and the responsibility adults have to bear for earlier disclosure of CSA to be possible. Our findings also shed light upon the dilemmas and complexity people face when considering and suspecting CSA, and the importance of addressing these dilemmas and help adults to act on their suspicions. One important implication was how information, the possibility of discussion and prevention programs can help in the process of disclosure of CSA, especially if targeting adults.

## Data Availability Statement

The original contributions presented in the study are included in the article/Supplementary Material, further inquiries can be directed to the corresponding author/s.

## Ethics Statement

The studies involving human participants were reviewed and approved by REK VEST (Norway). The patients/participants provided their written informed consent to participate in this study.

## Author Contributions

ES and JH participated in the design, data collection, analysis and writing. SS participated in the design, supervised data collection, and participated in analysis and writing. All authors contributed to the article and approved the submitted version.

## Conflict of Interest

The authors declare that the research was conducted in the absence of any commercial or financial relationships that could be construed as a potential conflict of interest.
