# Supplementary material for: What Do Survivors of Child Sexual Abuse Believe Will Facilitate Early Disclosure of Sexual Abuse?
Source: Front Psychiatry. 2021 Jun 14;12:639341. doi: 10.3389/fpsyt.2021.639341 (PMC8236530; doi:10.3389/fpsyt.2021.639341)
Supplement: Supplementary file 1 [file Data_Sheet_1.docx]

**Appendix: Interview Guide**

***Introduction:*** First of all I want to thank you for participating. As you know, everyone we interview have been exposed to child sexual abuse, and our focus is this interview is on the process leading to the discovery / disclosure of the abuse. We want to know more about your experiences and understanding of this process. The interview will be recorded on this digital voice recorder, and will then be transcribed. When transcribed the information will be anonymized, and the recording will then be deleted. We know that the focus in the interview can be very demanding and difficult to talk about. It is therefore important that you know that it is completely up to you what you choose to share, when you need breaks, or want to end the interview. In your experience, will it be ok to let me know when you need a break, or are there other ways I can understand that you need a break? Do you have any questions before we start?

| **Ok. First of all, can you tell me, in your own words, how the sexual abuse was discovered/ disclosed?** |
| --- |
| *Possible follow-up questions:*  What is important to know in order for me or others to understand your experiences?  How did you understand what happened to you at the time? Do you understand it differently now?  Do you think someone else understood that there were things going on that should not happen?  What do think contributed to you telling / not telling? In what way?  If disclosed because the participant told about the abuse: Do you have any thoughts on why you told about the abuse at that particular point in time?  Looking back, could anyone have done anything to make it easier for you to tell about the abuse? |
| **What happened after the abuse was discovered / disclosed?** |
| *Possible follow-up questions:*  How was it for you after the abuse was discovered/disclosed?  What was helpful to you?  Did something make it more difficult?  If in touch with health care system: Did you feel that the helpers you met understood you? |
| **Based on your experiences, what do you think is important in order to facilitate disclosure of child sexual abuse as early as possible?** |
| **Is there anything else I have not asked you, but you feel is important in order to understand the process you have been through in relation to the abuse being discovered / disclosed?** |

**Thank you so much for sharing your experiences, allowing us to learn more about this important topic!**
